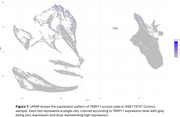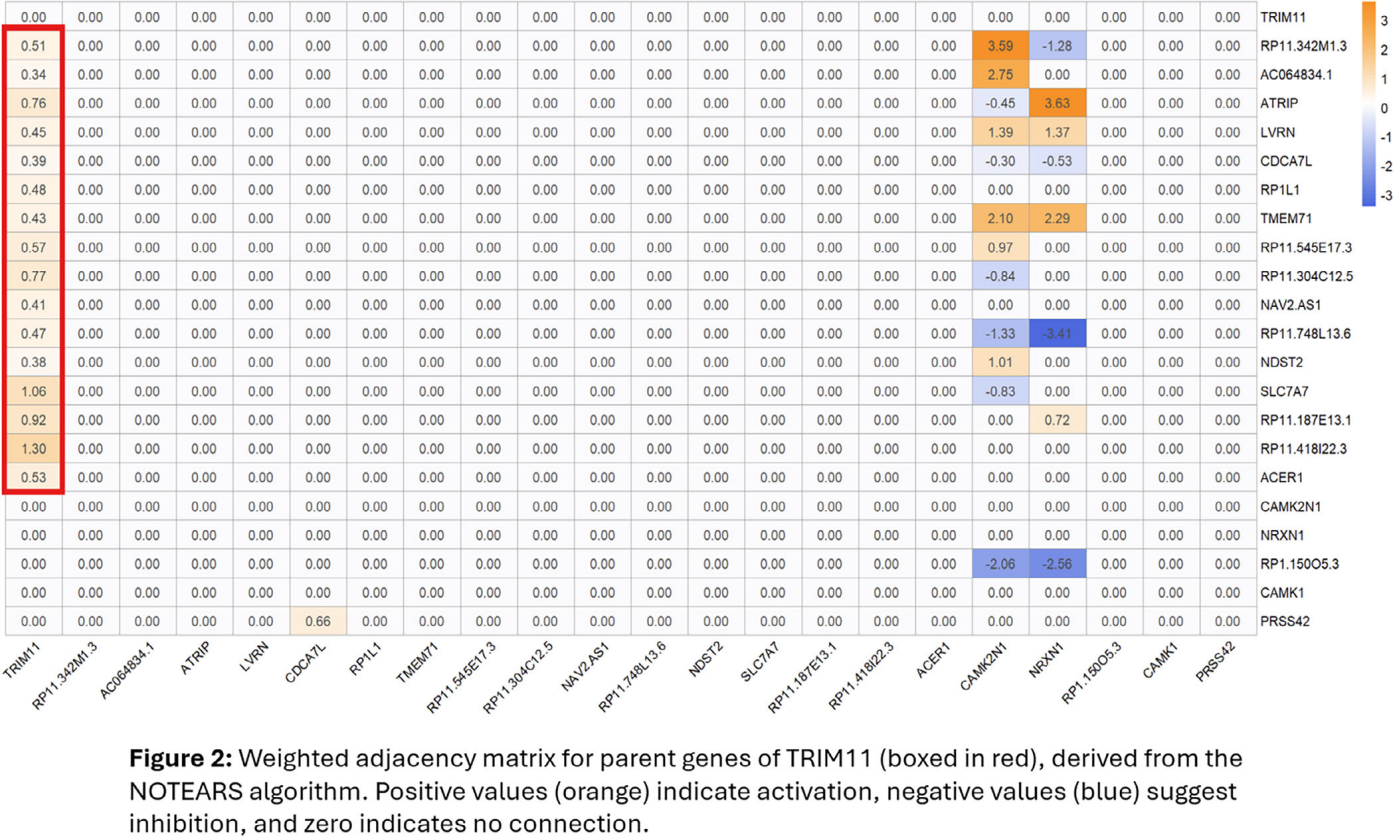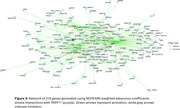# Causal Network of TRIM11: Exploring Druggable Pathways in Alzheimer's Disease

**DOI:** 10.1002/alz70855_106783

**Published:** 2025-12-24

**Authors:** Alvin Pham, Sumita Garai, Tianhua Zhai, Li Shen

**Affiliations:** ^1^ University of Texas at Austin, Austin, TX, USA; ^2^ University of Pennsylvania, Philadelphia, PA, USA; ^3^ Department of Biostatistics, Epidemiology, & Informatics, University of Pennsylvania, Philadelphia, PA, USA

## Abstract

**Background:**

It has been found that the Tripartite motif‐containing protein 11 (TRIM11) is downregulated in Alzheimer's Disease (AD). When overexpressed in AD mouse models, this gene is associated with the degradation of the amyloid tau protein and improving cognitive functions. The finding poses a promising pathway for potentially new targets that can upregulate TRIM11 expression and control AD symptoms. In this research, we employ single‐nucleus RNA sequencing (snRNA‐seq) data, Bayesian Networks, and Causal DAGs using the NOTEARS algorithm to identify potential genetic expression regulators of TRIM11, aiming to upregulate this gene in AD.

**Method:**

The snRNA‐seq dataset GSE173731 was obtained from the National Center for Biotechnology Information Gene Expression Omnibus (NCBI GEO), a publicly accessible database. This study focused on the control samples, consisting of 33,538 genes (coding and non‐coding) across 171,520 nuclei. Raw gene counts were processed in RStudio using the SingleCellExperiment and normalized via Relative Log Expression (RLE). Gene‐gene interactions were analyzed using a three‐step framework: Pearson correlation assessed linear associations of other genes with TRIM11, Bayesian analysis identified probabilistic dependencies, and the NOTEARS algorithm refined causal inference with a sparse, acyclic network.

**Result:**

After calculating the Pearson correlation of all other genes to TRIM11, 1,088 genes were found to be positively correlated to TRIM11, with a significant‐adjusted *p*‐value threshold of 1.47x10‐6, determined by the Bonferroni Correction. Applying the Bayesian framework to those 1,088 genes, we identified 219 genes positively influencing TRIM11. The NOTEARS algorithm was then applied to these 219 genes, of which 16 genes were found to have a direct, positive causal relationship with TRIM11, suggesting their potential regulatory or functional roles in its expression. Examples of these genes include ATRIP (plays a key role in DNA damage response), NDST2 (involved in heparan sulfate biosynthesis), and SLC7A7 (critical for amino acid transport).

**Conclusion:**

By integrating Pearson correlation analysis, Bayesian framework, and NOTEARS causal network modeling, we identified key genes that may influence TRIM11 expression. Future studies can focus on experimental validation of these genes and exploring their functional roles in pathways associated with TRIM11 to uncover potential therapeutic targets.